# Fungal Melanonychia as a Solitary Black Linear Vertical Nail Plate Streak: Case Report and Literature Review of Candida-Associated Longitudinal Melanonychia Striata

**DOI:** 10.7759/cureus.14248

**Published:** 2021-04-01

**Authors:** Philip R Cohen, Joseph Shurman

**Affiliations:** 1 Dermatology, San Diego Family Dermatology, National City, USA; 2 Pain Management/Palliative Care, Scripps Memorial Hospital, La Jolla, USA

**Keywords:** black, candida, fungal, linear, longitudinal, melanonychia, nail, solitary, striata, vertical

## Abstract

Longitudinal melanonychia striata, presenting as a black linear vertical band of the nail plate, can be caused by pigmented lesions and non-pigmented etiologies. A fungal infection of the nail plate, also referred to as onychomycosis or tinea unguim, can result from dermatophytes, non-dermatophyte molds, and *Candida*. Albeit rare, *Candida*-associated fungal melanonychia can present as a longitudinal black nail plate streak. The case of a 79-year-old man who developed a solitary linear black streak on his right fourth fingernail after a prior history of recent trauma to the digit’s nail folds is described; the fungal culture grew *Candida parapsilosis*. Including our patient, *Candida*-associated longitudinal melanonychia striata has been described in four women and two men ranging in age from 40 to 79 years (median, 70 years) at diagnosis. The black streak, present from one month to one year (median, seven months), affected either a hand digit (five patients) or the great toe (one patient). Fungal organisms were visualized on either a potassium hydroxide preparation (one patient), pathologic evaluation of a nail plate specimen (three patients), or both (one patient). Culture grew *Candida parapsilosis* (two patients), *Candida* *species* (two patients), *Candida albicans* (one patient), and *Candida tropicalis* (one patient). All of the patients experienced clinical improvement after treatment. Topical treatment (5% amorolfine hydrochloride nail lacquer for two patients or modified Castellani paint and 1% clotrimazole cream for one man) or oral itraconazole (either as monotherapy for two women or combined with 5% amorolfine hydrochloride nail lacquer for one woman) was successfully used. Although the clinical presentation of fungal melanonychia can mimic subungual melanoma when it appears as a solitary black linear vertical nail plate streak, investigative studies--such as a potassium hydroxide preparation, nail plate pathology, nail matrix biopsy, and/or fungal culture--can be used to establish the diagnosis of *Candida*-associated longitudinal melanonychia striata and exclude the diagnosis of a pigmented melanocytic tumor.

## Introduction

Melanonychia is a black nail. Longitudinal melanonychia striata is a black linear vertical band that extends from the proximal nail fold to the distal end of the nail plate. There are several etiologies for longitudinal melanonychia striata, including pigmented lesions and non-melanocytic causes, such as infections, medications, and systemic diseases [1,2].

Onychomycosis is a fungal infection of the nail plate and is usually caused by a dermatophyte organism. Common clinical presentations of the nail plate’s dermatophyte tinea infection include distal lateral subungual onychomycosis, proximal subungual onychomycosis, and superficial white onychomycosis. However, *Candida* and non-dermatophyte molds can also cause fungal infection of the nail plate; although these usually present as yellow or white discoloration, they can also, albeit less commonly, present as melanonychia [3,4].

A man with a solitary linear black streak on a single fingernail presented for evaluation; he had a prior history of recent trauma to the digit’s proximal and lateral nail folds. A fungal culture grew *Candida parapsilosis*. *Candida* species-associated longitudinal melanonychia striata is uncommon; the features of our patient and other individuals with the new onset of a black linear nail plate streak caused by *Candida* species are reviewed [5-8].

## Case presentation

A 79-year-old man presented for evaluation of a new vertical black streak on the nail of his right fourth fingernail. Four months earlier, he had a manicure of both hands’ thumbs and fingers; the manicurist used a firm instrument to manipulate all of his cuticles. He subsequently developed painful red swelling of the proximal and lateral nail folds on his right fourth digit.

Several clinicians evaluated his acute paronychia. He was empirically treated topically with gentamicin sulfate 0.1% ointment and clotrimazole 1.0% cream; the tender erythema subsequently resolved, and treatment was stopped after two weeks. An aerobic bacterial culture and a fungal culture eventually grew few *Serratia marcescens* and *Candida albicans*, respectively; an anaerobic culture did not isolate any anaerobes.

**Figure 1:**
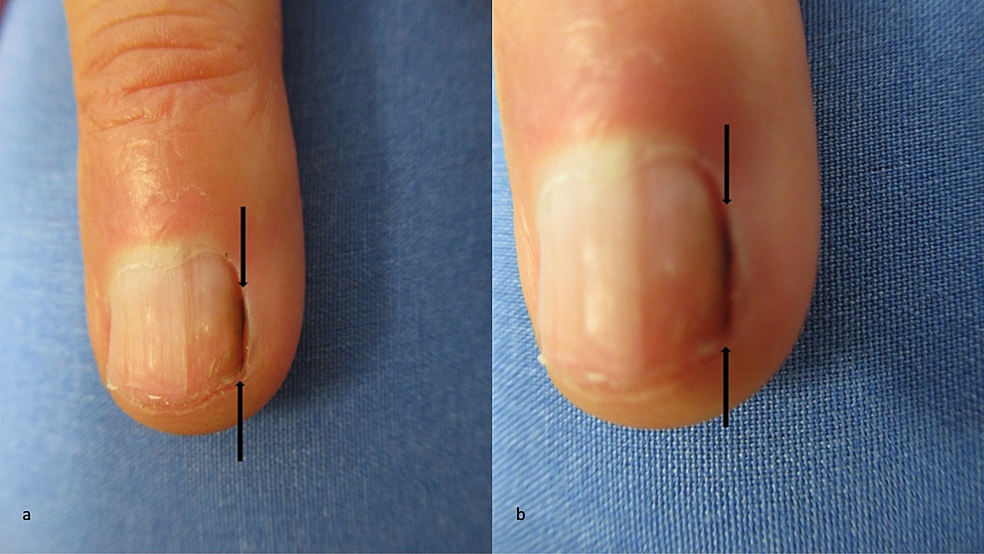
Candida parapsilosis-associated longitudinal melanonychia striata Distant (a) and closer (b) views of the right fourth distal finger of a 79-year-old man show a solitary black linear vertical nail plate streak (located between the tips of the black arrows). Culture of the fungal melanonychia grew *Candida parapsilosis*.

During the next three months, he developed an asymptomatic pigmented streak on his right fourth fingernail. Cutaneous examination of the nails on his thumbs and fingers showed a 1-mm linear black band on his right fourth finger. It extended from the proximal portion of the lateral nail fold closest to his thumb to the distal tip of his nail (Figure 1).

The clinical presentation was suggestive of a diagnosis of longitudinal melanonychia striata. He was treated topically twice daily with modified Castellani paint (a solution that consists of phenol, acetone, resorcinol, alcohol, and water) and 1% clotrimazole cream; both medications were applied to the affected nail plate and the adjacent lateral nail fold. An aerobic bacterial culture only demonstrated normal skin flora; however, the fungal culture grew *Candida parapsilosis*.

**Figure 2:**
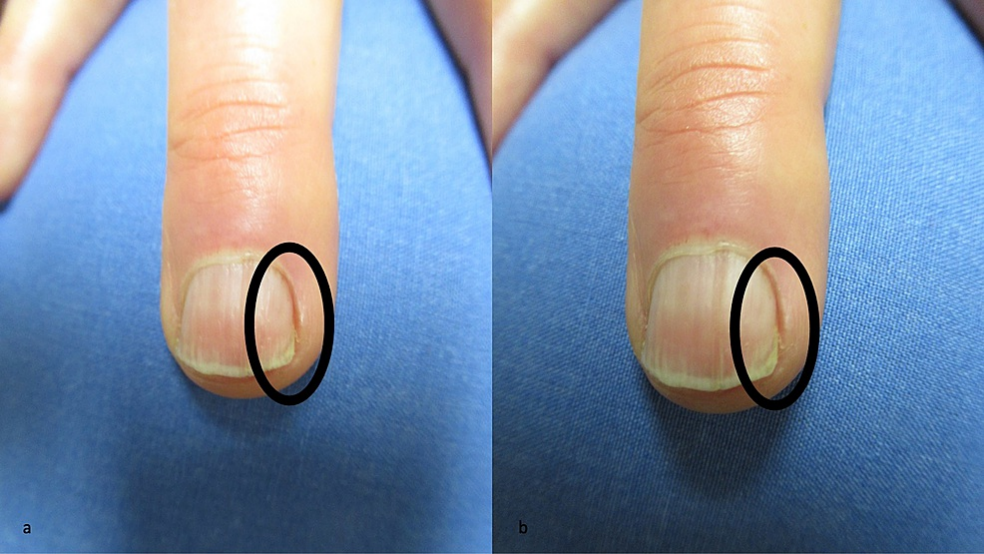
Complete resolution of Candida parapsilosis-associated longitudinal melanonychia striata Distant (a) and closer (b) views of the right fourth distal finger demonstrate complete clearing of the linear fungal melanonychia (which had been present in the area within the black oval) after three months of twice daily topical treatment with modified Castellani paint (a solution that consists of phenol, acetone, resorcinol, alcohol, and water) and 1% clotrimazole cream.

The right fourth fingernail began to show normal-appearing proximal nail plate growth. The pigmented streak completely cleared after three months of daily treatment and the agents were discontinued. Follow-up evaluation of his right fourth fingernail, two months after stopping the medications, showed a normal nail plate without black dyschromia (Figure 2).

## Discussion

Longitudinal melanonychia striata usually presents from either melanocytic proliferation or melanocytic activation in the nail matrix. The nail matrix is a melanocyte-containing germinative epithelium that produces the nail plate. Activated melanocytes, within the nail matrix, transfer melanin-rich melanosomes distally into the nail plate cells, which clinically present as a black linear vertical band [1,2].

Melanocytic proliferation is observed with benign and malignant pigmented lesions. Melanocytic activation is associated with many etiologies, including chronic local trauma, dermatologic conditions, endocrine disorders, iatrogenic causes, infection, medications, nutritional deficiencies, pregnancy, and being related racially. In some circumstances of fungal melanonychia, the organisms (such as *Candida albicans*) can synthesize melanin in the yeast cells [1,2,9].

*Candida* is a yeast that can be associated with onychomycosis, more commonly affecting fingernails. *Candida albicans* infection can appear as a white, potentially dystrophic, nail plate. *Candida parapsilosis* has been associated with onychomycosis that has presented as either white, yellow, or dull gray nails [10-14].

Albeit uncommon, *Candida* species can cause melanonychia. Diffuse black nails have been seen due to *Candida albicans*, *Candida humicola*, and *Candida parapsilosis* [15-18]. Rarely, *Candida* species have been associated with a linear black streak of the nail plate; the pathogenesis for the development of a vertical linear streak in these individuals remains to be determined [1,2].

*Candida* nail plate infection is included in the differential diagnosis of causes of longitudinal melanonychia striata. Indeed, it is listed as a potential etiology in most review articles on the subject [1,2]. However, to the best of our knowledge, including our patient, *Candida* species-associated longitudinal melanonychia striata has only been described in four women and two men (Table 1) [5-8]. 

**Table 1:** Clinical features and investigative studies of patients' Candida species-associated longitudinal melanonychia striata Abbreviations: A, age (years); AHNL, 5% amorolfine hydrochloride nail lacquer; Bx, biopsy; C, case; CI, clinical improvement; Clot, 1% clotrimazole cream; CR, current report; d, day; DD, daily dose; Dur, duration (months); F, finger; Fup, follow-up; G, gender; Ko, Korean; KOH, potassium hydroxide preparation; L, left; M, man; MCP, modified Castellani paint; mg, milligrams; Na, nationality; ND, not done; NI, no improvement; NS, not stated; q, each; R, right; SysTx, systemic treatment; T, toe; TopTx, topical treatment; W, woman; wk, week; x, for; 1, first (finger = thumb, toe = great toe); 2x, twice; 3, third; 4, fourth; +, positive; -, negative; /, per. ^a^Positive KOH preparations showed spores, pseudohyphae, and/or round yeast [6,7]. ^b^Specimens of nail plate or nail bed stained with hematoxylin and eosin or period-acid Schiff predominantly showed spores; occasionally budding yeast or pseudohyphae were also observed [5,7,8]. ^c^Two KOH preparations, at three-month intervals, were done; only the first was positive for scanty round yeasts. ^d^The fungal organism was identified using an automated biochemical test kit: the Vitek 2 ID-YST system (bioMerieux, Marcy-l'Étoile, France). ^e^*Candida albicans* was initially cultured from the affected digit during the acute paronychia; however, *Candida parapsilosis* was cultured when he subsequently presented with a linear black band of fungal longitudinal melanonychia striata.

C	A Na G	Site	Dur	KOH^a^	Bx^b^	Culture	TopTx Drug Dose Dur	SysTx Drug DD Dur	Fup	Ref
1	49 Ko W	R1F	5	-	+	Candida tropicalis	None	Terbinafine 250 mg 1 Itraconazole 200 mg 4	NI CI	[5] C3
2	55 Ko W	R1F	1	+	ND	*Candida* species	AHNL 2x/wk 3	None	CI	[6]
3	60 Fr M	L3F	12	+/-^c^	+	Candida parapsilosis	AHNL weekly 10	None	CI	[7]
4	61 Ko W	L1T	8	-	+	*Candida* species	None	Itraconazole 200 mg 3	CI	[5] C2
5	65 Ko W	L1F R1F	12	-	+	*Candida albicans*^d^	AHNL NS NS	Itraconazole 400 mg x 7d q mon x 3	CI	[8]
6	79 Am M	R4F	3	ND	ND	*Candida parapsilosis*^e^	MCP 2x/d 3 Clot 2x/d 3	None	CI	CR

The women with *Candida* species-associated longitudinal melanonychia striata ranged in age from 49 to 65 years (median, 58 years) at diagnosis. The men ranged in age from 60 to 79 years (median, 61 years) when their *Candida* longitudinal melanonychia striata was diagnosed. Overall, the six patients ranged from 40 to 79 years (median, 70 years) [5-8].

All of the women were Korean. In contrast, one of the men was French. Our patient was American [5-8].

A digit of the hand was affected in five of the patients; the great toe was involved in one of the women. Indeed, the thumb or the great toe was the site of fungal melanonychia in four of the patients; both thumbs were affected in one woman. Either the third or the fourth finger was involved in the two men [5-8]. 

There was no predilection for the *Candida* infection to localize to a specific side of the body. The right side was affected in four patients, and the left side was involved in three patients. One woman’s fungal melanonychia occurred on both of her thumbnails [5-8].

Trauma to the affected digit had occurred in our patient and the other man [7]. Our patient suffered an injury to his nail folds during a manicure; this resulted in an acute paronychia and likely predisposed him to the development of his fungal melanonychia. The 60-year-old man experienced an open fracture of the distal phalanx of the affected finger resulting from a crush injury 37 years earlier. Subsequently, the man developed a chronic dystrophy of his digit’s lateral nail that persisted after the operative repair of the finger and was the site of his *Candida*-associated longitudinal melanonychia striata [7]. There was no history of trauma for two of the women [6,8]. The occurrence of prior injury to the affected nail was absent in the reports of the remaining patients [5].

The duration of the linear black streak before establishing the diagnosis of *Candida* longitudinal melanonychia striata ranged from one to 12 months (median, seven months). Until a diagnosis was confirmed, the range of time was one to 12 months for the women (median, seven months). A similar duration of time before documenting the diagnosis was observed in the men: the range was three to 12 months (median, eight months) [5-8].

A potassium hydroxide preparation of scrapings from the affected nail plate was performed for five of the patients. One woman had a positive initial evaluation and second testing three months later that was negative. A second woman also had a positive examination. The positive potassium hydroxide preparation demonstrated either numerous hyaline pseudohyphae and spores with amorphous brownish pigments surrounding them or scanty round yeasts and no mycelia [6,7]. The three patients whose potassium hydroxide preparation was negative did not show any fungal elements [5,7,8].

A piece of the affected nail plate from four patients was sent for pathologic evaluation. The stained specimens with hematoxylin and eosin showed refractile pseudohyphae or ovoid spores or both in the nail bed stratum corneum or round budding yeasts with no mycelial elements [5,7]. The stained specimens with periodic-acid Schiff showed numerous spores or a few pseudohyphae or both in the stratum corneum, or numerous ovoid spores in the nail plate, or round budding yeasts with no mycelial elements [5,7,8]. 

Culture of the affected nail plate or nail fold was performed. The most common fungal organisms were *Candida parapsilosis* (two patients) and *Candida* *species* (two patients). The cultures from the other women demonstrated either *Candida albicans* (one patient) or *Candida tropicalis* (one patient) [5-8].

All of the patients showed improvement in their nail plate fungal melanonychia following treatment. The patients received either topical treatment (two men and one woman), systemic treatment (two women), or topical and systemic treatment (one woman). One woman who was successfully treated with systemic itraconazole initially did not show any improvement after a month of oral terbinafine [5-8].

Itraconazole was uniformly efficacious in the management of longitudinal melanonychia striata caused by *Candida* infection. Two women received 200 mg daily of itraconazole for either three or four months [5]. The third woman received three monthly pulses (each consisting of 400 mg of itraconazole for seven days) in addition to topical therapy with 5% amorolfine hydrochloride nail lacquer [8].

*Candida*-associated fungal longitudinal melanonychia striata in two of the other patients was also successfully treated topically with 5% amorolfine hydrochloride nail lacquer monotherapy. A 55-year-old woman underwent application of the lacquer twice weekly for three months [6]. The other patient, a 60-year-old man, applied the antifungal agent weekly for 10 months [7].

Our patient was successfully treated with twice-daily topical application of modified Castellani paint and 1% clotrimazole cream. The original formulation of Castellani paint included fuchsin (basic or carbol), alcohol, boric acid, phenol, acetone, resorcinol, and water. The modified version is colorless since the carbolfuchsin is not included. Also, in the current formulation of Castellani paint and modified Castellani paint, the boric acid (which is a potential carcinogen) is absent [19,20].

The fuschin in Castellani paint is very active against *Candida*. However, the phenol and resorcinol in Castellani paint and modified Castellani paint have antimicrobial activity. Other modified Castellani paint properties, such as its drying and keratolytic activity, also seem to result in its clinical effectiveness when treating *Candida* infections [19,20]. We are not aware of any additional variants of Castellani paint that are under investigation for treating *Candida* infection.

## Conclusions

*Candida*-associated fungal melanonychia can present as a longitudinal black nail plate streak. *Candida*-associated longitudinal melanonychia striata has been described in four women and two men. *Candida parapsilosis* (two patients), *Candida* *species* (two patients), *Candida albicans* (one patient), and *Candida tropicalis* (one patient) grew from the fungal culture of the affected nail plate or nail fold. All of the patients improved clinically after either topical therapy or a systemic antifungal or both. *Candida*-associated longitudinal melanonychia striata can mimic subungual melanoma when it presents as a solitary black linear vertical nail plate streak; however, investigative studies--such as a potassium hydroxide preparation, nail plate pathology, nail matrix biopsy, and/or fungal culture--can be used to exclude a pigmented melanocytic tumor and establish the diagnosis of a fungal melanonychia.
